# Altered Pulmonary Glucose Transport Is Restored by Metformin Treatment in an Obese Type 2 Diabetic Animal Model

**DOI:** 10.3390/metabo15110717

**Published:** 2025-11-02

**Authors:** Allison Campolo, Zahra Maria, Véronique A. Lacombe

**Affiliations:** 1 Department of Physiological Sciences, College of Veterinary Medicine, Oklahoma State University, Stillwater, OK 74078, USA; 2 Department of Biochemistry and Physiology, University of Oklahoma Health Sciences Center, Oklahoma City, OK 73104, USA

**Keywords:** glucose transporter, GLUT trafficking, hyperglycemia, obesity, metabolism, lung, mice

## Abstract

**Background/Objectives:** Obesity and hyperglycemia predispose patients to respiratory infections. Although the lung is a major organ to utilize glucose, pulmonary glucose homeostasis in type 2 diabetic (T2Dx) subjects remains poorly characterized. We hypothesized that pulmonary glucose transport would be altered during T2Dx, which would be rescued with long-term metformin treatment. **Methods:** T2Dx was induced by feeding mice a high-fat diet for 16 weeks, with metformin treatment administered during the final 8 weeks. **Results:** Glucose transporter (GLUT) protein expression and trafficking was quantified by Western blotting and the biotinylated photolabeling assay, respectively. T2Dx mice exhibited obesity, and increased glucose levels in blood and bronchoalveolar lavage (BAL) fluid. T2Dx also significantly decreased protein expression of GLUTs from Class I (i.e., GLUT-2 and -4) and class III (i.e., GLUT-10 and -12) isoforms in lung. Metformin treatment restored the protein expression of GLUT-2, -4, and -10, but not GLUT-12. Pulmonary cell surface expression of GLUT-4 and -8 was also significantly reduced in T2Dx mice and rescued by metformin. **Conclusions:** These findings suggest that alterations in pulmonary GLUT expression and trafficking during diabetes could contribute to the elevated airway glucose levels and severity of respiratory infections. Metformin treatment restored pulmonary glucose transport during T2Dx.

## 1. Introduction

Type 2 diabetes mellitus (T2Dx) affects over 90% of individuals with diabetes worldwide and is characterized by insulin resistance, hyperglycemia, weight gain and low-grade chronic inflammation [1,2]. Although T2Dx increases the risk of cardiovascular, renal, and neurological complications, its impact on pulmonary function and susceptibility to respiratory infections is now becoming increasingly recognized [3,4,5]. For instance, epidemiological studies have linked poor glycemic control with a higher incidence and severity of respiratory infections, including pneumonia, tuberculosis, and asthma [3,4,5,6]. However, the mechanisms underpinning this increased susceptibility remain incompletely understood.

Glucose homeostasis in the lung plays a critical role in maintaining airway epithelial barrier integrity and immune function. A relatively constant ratio between blood glucose and airway surface liquid (ASL) glucose concentrations is maintained under normal conditions [7]. Type 2 diabetes is due to a lack of insulin action with secondary impairment in glucose transport and utilization in insulin-sensitive tissue (e.g., striated muscle and adipose tissue). Hyperglycemia leads to increased glucose concentration in the ASL to ∼1–3 mM, which can impair airway defenses and promote infection [7,8,9]. However, despite the lung’s reliance on glucose transport, studies on the regulation of glucose transport in healthy versus diabetic lung remain sparse.

Glucose transport is regulated by a family of specialized proteins called glucose transporters (GLUTs) that facilitate glucose movement across cellular membranes. There are currently 14 known glucose transporters (GLUTs), divided into 3 classes. As part of the class I, GLUT1 is the predominant basal glucose transporter while GLUT4, the predominant insulin-sensitive glucose transporter, translocates from an intracellular pool to the plasma membrane to enhance glucose transport in insulin-sensitive tissue [10,11,12,13,14]. GLUT8, a novel class III isoform, plays a role in both cellular glucose and fructose uptake from the blood stream into many tissues, including in the heart and the lung [10,11,15]. Importantly, this isoform may be a novel therapeutic target for metabolic diseases. Alongside GLUT2, GLUT10 has been shown to be an important basal transporter for glucose uptake between the airway and the bronchial epithelium in both humans and rodents [9,15]. GLUT12, a novel class III isoform, has been found to be expressed in many tissues, including ionocytes and ciliated pulmonary cells, and primarily functions as basal GLUT located at cell surface in the heart [9,15,16,17]. It has been hypothesized that GLUTs regulate pulmonary glucose uptake and ASL glucose levels [7,9]. Although it is well established that obesity and insulin resistance alter GLUT expression and/or trafficking in insulin-sensitive tissue [11,13,14,16], their pathophysiological effects on pulmonary glucose transport are unknown.

Metformin, a first-line therapy for T2Dx, primarily acts by enhancing insulin sensitivity, promoting glucose uptake in insulin-sensitive tissues, and reducing hepatic gluconeogenesis [18]. In peripheral tissues such as skeletal muscle and adipose tissue, metformin improves GLUT4 trafficking and function [18,19]. Its potential effects on pulmonary glucose transport, however, are less understood.

In this study, we sought to characterize the alterations in pulmonary GLUT isoform expression during T2Dx and to evaluate whether metformin treatment could reverse these changes. We hypothesized that T2Dx would lead to reduced GLUT expression and trafficking in the lung, and that metformin would partially or fully rescue these defects.

## 2. Materials and Methods

### 2.1. Obese Type 2 Diabetic Animal Model

Healthy male 16-week-old C57Bl/6 mice (Charles River, Wilmington, MA, USA) were fed with either regular chow (AIN-93M Mature Rodent Diet, product #D10012M, Research Diets, New Brunswick, NJ, USA) or a high-fat diet (Rodent Diet with 60% kcal from fat, product #D12492, Research Diets, Brunswick, NJ, USA) for 16 weeks in order to induce obesity and T2DX. A subset of both the healthy mice and the high-fat diet-fed mice received metformin treatment for an additional 8-week period. Metformin was added to their water at a rate of 200 mg/kg/day. A total of 24 mice finished the study (6 mice per group) for the in vivo measurements. To qualify for the study, mice had to demonstrate a normal resting blood glucose level and no other underlying health conditions. Mice would have been removed from the study at any point if they demonstrated signs of pain or developed health conditions not related to a high-fat diet. Mice were inspected at least daily for general condition. No mice were removed from this study. All mice were considered equal after initial health screenings were performed and were randomly numbered to be assigned to treatment groups. Due to the nature of the high-fat diet (which is blue and produces obese mice, and control mice cannot be housed with high-fat mice) and the metformin administration (which is administered in the drinking water in the cage, further preventing sharing of cages between groups), it was not possible to randomize this study or blind examiners from treatment groups. The same unblinded examiner which determined the groups took the measurements and performed the initial data analysis. Throughout the study, the data was reviewed by blinded collaborators. For the entirety of the study, mice were housed in and cared for by the laboratory animal research building at Oklahoma State University (Institutional Animal Care and Use Committee protocol # VM-18-3).

### 2.2. Bronchoalveolar Lavage (BAL) Fluid Analysis

Mice were anesthetized using 3% isoflurane. A tracheal cannula was inserted, and 1 mL of chilled, sterile phosphate-buffered saline (PBS) was instilled into the lungs and retrieved. This lavage was repeated three times to collect a total of 2–3 mL of BAL fluid per animal. Glucose concentrations in the BAL fluid were measured spectrophotometrically using the Amplex Red Glucose Assay Kit (Thermo Fisher #A22189, Waltham, MA, USA).

### 2.3. Photolabeling Biotinylation Assay

Briefly, lung homogenates were incubated with the biotinylated bis-glucose photolabeling reagent (Toronto Research Chemicals, Toronto, ON, Canada), which specifically interacts with the extracellular binding site of GLUTs upon photochemical reaction (Rayonet photochemical reactor, Southern New England UV). Crude membrane protein extracts were obtained following ultracentrifugation (227,000× *g*, 90 min at 4 °C). Recovery of photolabeled cell surface glucose transporters was achieved using streptavidin isolation (bound to 6% agarose beads, Thermo Fisher, product #20349, Waltham, MA, USA) to separate intracellular GLUTs (“unlabeled”) from cell-surface GLUTs (“labeled”). Proteins from the unlabeled and the labeled fractions were quantified by Western blotting, as previously described [11,12,15,17].

### 2.4. Western Blotting

Whole lung tissues were lysed to obtain total protein extracts, following protocols established in prior studies [15]. Protein concentrations were determined using the bicinchoninic acid (BCA) assay (Thermo Fisher, Cat. #23227, Waltham, MA, USA), and samples were prepared for Western blot analysis as previously outlined [11,12,13,15,16,17]. Primary antibodies used included: polyclonal rabbit anti-mouse GLUT1 (1:500, Abcam #ab652), GLUT2 (1:500, Santa Cruz #sc-7582), GLUT3 (1:500, Abcam #ab54460), GLUT4 (1:750, AbD Serotec #4670-1704), GLUT8 (1:500, Bioss #bs4241R), GLUT10 (1:750, Thermo Fisher #PA1-46137), and GLUT12 (1:500, Abcam #ab75441). β-actin was detected using a mouse monoclonal antibody (Santa Cruz #sc-47778). Secondary antibodies conjugated to horseradish peroxidase (HRP) were diluted in TPBS containing 5% milk. These included: donkey anti-rabbit IgG H&L (1:2500, Abcam #ab7083) for GLUT1, GLUT2, GLUT10, and GLUT12; anti-rabbit IgG (1:4000, Santa Cruz #sc-2020) for GLUT3; donkey anti-rabbit HRP-linked (1:3000, GE Healthcare #NA934) for GLUT4 and GLUT8; and mouse IgG kappa binding protein (1:5000, Santa Cruz #sc-516102) for β-actin. Detection was performed using enhanced chemiluminescence (SuperSignal Max Sensitivity Substrate, Thermo Fisher #34095). Band intensities and molecular weights were analyzed using GelPro Analyzer software version 4.5 (Media Cybernetics, Rockville, MD, USA). To confirm equal protein loading, membranes were stripped (Thermo Fisher #21063) and reprobed with β-actin. Protein levels of the class I and class III GLUTs were normalized to the loading control and its respective controls.

### 2.5. Immunohistochemistry

Whole lung tissues were perfused with paraformaldehyde and fixed in formalin for no more than 48 h prior to sectioning. Samples were embedded in paraffin and cut into 4 µm sections, then stored at −20 °C until further processing. Slides were deparaffinized and rehydrated through a series of washes: three 5 min xylene incubations, followed by two 10 min incubations in 100% ethanol, two 10 min incubations in 95% ethanol, and two 5 min rinses in distilled deionized water. After a 5 min wash in 1× TBST, slides were blocked at room temperature for one hour using 5% goat serum in TBST. Primary antibody incubation was performed overnight at 4 °C using anti-GLUT4 (BioRad #4670-1704, Hercules, CA, USA). Slides were then treated with Nova Red substrate (Vector Laboratories #SK-4800, Burlingame, CA, USA) for 15 min, followed by a 10 s counterstain with Hematoxylin QS (Vector Laboratories #H-3404-100). After rinsing with distilled deionized water for 1–2 min, slides were dehydrated and coverslipped.

### 2.6. Statistical Analyses

Each mouse served as an experimental unit. Normality for one-way ANOVA measurements was assessed via the Shapiro-Wilks test and determined to be normally distributed. Two-way repeated measure analysis of variance (ANOVA, treatment and time factors) for the in vivo measurements, and a one-way ANOVA (treatment factors) for the in vitro measurements were performed, using a statistical software package (SigmaPlot 11.0, Systat Software, Inc, San Jose, CA, USA) as appropriate. In vivo data was not found to possess a normal distribution and so was analyzed via a post hoc Mann–Whitney U test and pursuing Benjamini, Krieger, and Yekutieli method to correct for multiple comparisons (false discovery rate set at 1%). Correlations were analyzed by linear regression. Statistical significance was defined as *p* < 0.05 for all comparisons.

## 3. Results

### 3.1. Hyperglycemia and Obesity in Type 2 Diabetic Mice

**Figure 1:**
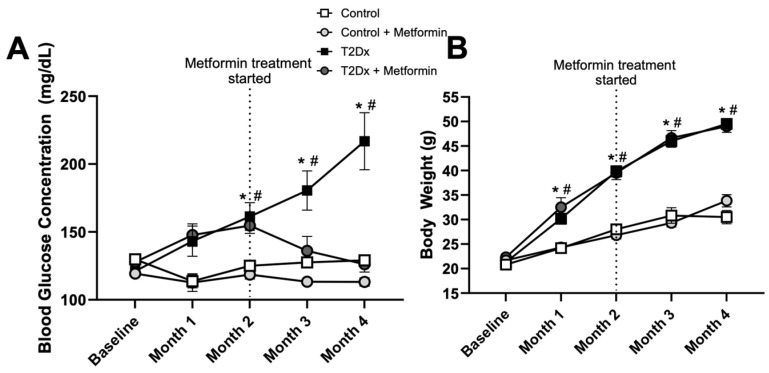
Hyperglycemia was rescued by metformin treatment, without affecting body weight, in obese type 2 diabetic (T2Dx) animals. (**A**) Mean ± SE of fasted serum blood glucose levels of untreated and treated T2Dx and control groups. (**B**) Mean ± SE of body weight of untreated and treated T2Dx and control groups. n = 6/group. * *p* < 0.05 vs. control, # *p* < 0.05 vs. baseline, via two-way repeated measure ANOVA.

Mice fed a high-fat diet (with 60% kcal from fat) for 16 weeks displayed higher blood glucose concentration vs. baseline (*p* < 0.005) and control counterparts (*p* < 0.005 for months 2–4), indicating that these mice were diabetic. At 8 weeks, subsets of both control and diabetic mice were treated with metformin for an additional 8 weeks, which significantly reduced blood glucose concentration of T2Dx to a normal level (Figure 1A). Mice fed a high-fat diet for 16 weeks also displayed obesity, with an increased body weight by ~220% at 4 months (*p* < 0.005 vs. baseline). Metformin treatment did not alter body weight in healthy and T2Dx mice (Figure 1B).

### 3.2. Increased Glucose Levels in BAL Fluid of Diabetic Obese Mice

**Figure 2:**
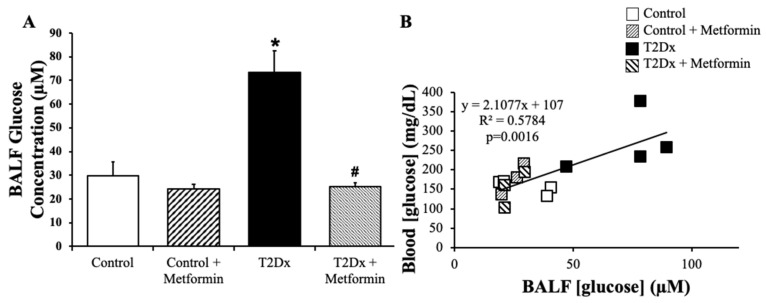
Obese type 2 diabetic (T2Dx) exhibited higher glucose concentration in the airway, which was rescued with metformin treatment. (**A**) Mean ± SE of glucose concentrations in bronchoalveolar lavage fluid (BALF) of untreated and treated T2Dx and control groups (n = 3–4/group); * *p* < 0.05 vs. Control, # *p* < 0.05 vs. Diabetic, via two-way ANOVA. (**B**) Linear correlation between blood and BAL fluid glucose levels.

In order to determine whether TD2x also affects airway glucose homeostasis, glucose concentration was measured in BAL fluid via spectrophotometric assay. Obese TD2x mice exhibited significantly higher glucose concentrations in BAL fluid versus their control counterparts (*p* = 0.028). This was rescued by long-term treatment with metformin (Figure 2A). As for blood glucose levels, metformin treatment did not affect glucose concentration in BAL fluid of healthy mice. Blood glucose levels were significantly positively correlated with BAL fluid glucose concentrations in TD2x mice (*p* = 0.0016, Figure 2B).

### 3.3. Type 2 Diabetic Whole Lung GLUT Protein Expression and Trafficking

**Figure 3:**
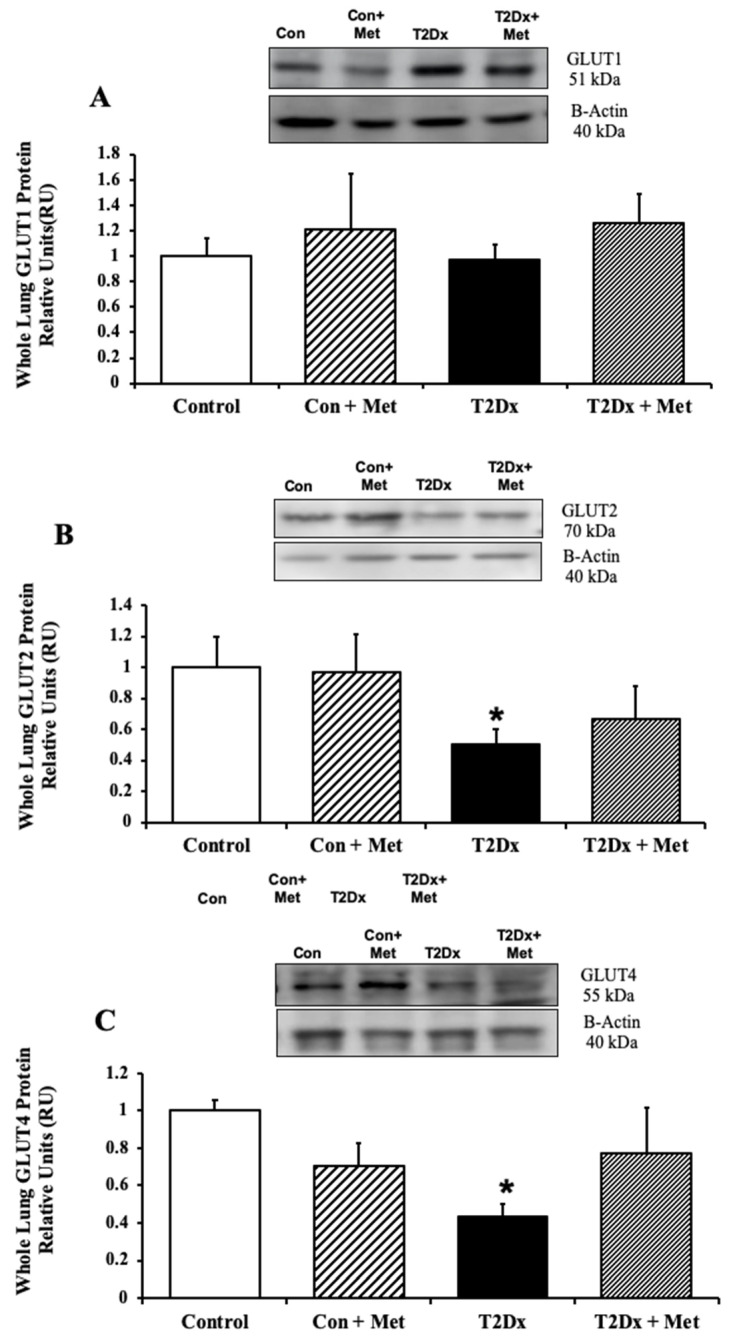
Type 2 diabetes (T2Dx) induced decreased pulmonary protein expression of the class I glucose transporter (GLUT), which was partially rescued by in vivo metformin (Met) treatment. Total protein expression of (**A**) GLUT1, (**B**) GLUT2, and (**C**) GLUT4 in the whole lung of control (con), untreated T2Dx, and metformin-treated T2Dx mice. Top panels: representative Western blot from total lysate of the whole lung; loading control: beta actin. Bottom panels: Mean ± SE of total GLUT protein content (values normalized to β-actin and its respective controls, n = 4–6/group). * *p* < 0.05 vs. control. Methods: Western blotting.

**Figure 4:**
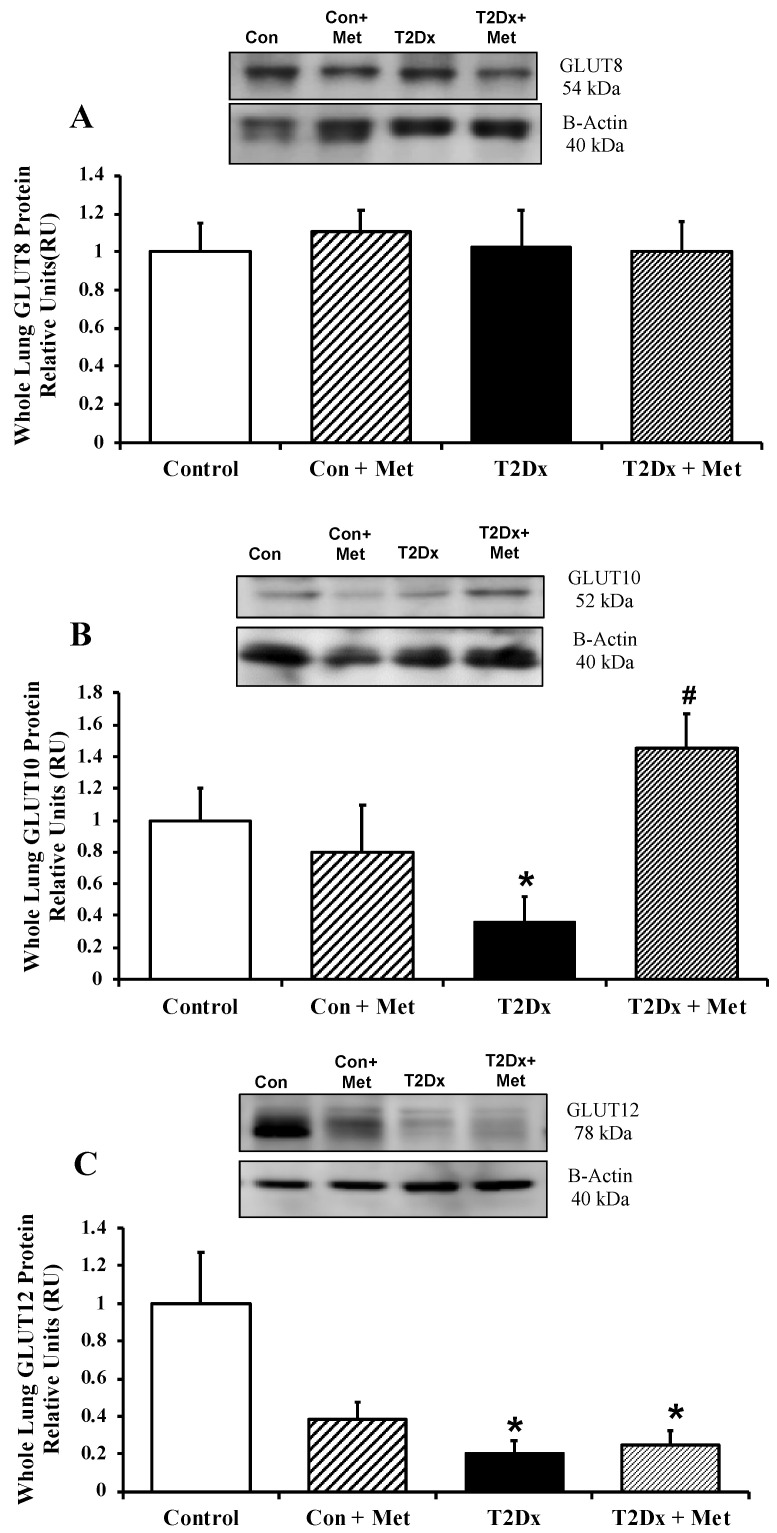
Type 2 diabetes (T2Dx) decreased pulmonary protein expression of the class III glucose transporter (GLUT), which were rescued by in vivo metformin (Met) treatment. Total protein expression of (**A**) GLUT8, (**B**) GLUT10, and GLUT12 (**C**) in the whole lung of control (Con), untreated T2Dx, and metformin-treated T2Dx animals. Top panels: representative Western blot of the class III GLUT and β-actin (loading control). Bottom panels: Mean ± SE of total GLUT protein content (values normalized to β-actin and its respective controls, n = 4–6/group). * *p* < 0.05 vs. control, # *p* < 0.05 vs. T2Dx via one-way ANOVA. Methods: Western blotting.

In order to assess whether alterations in airway glucose homeostasis could alter pulmonary glucose transport, protein expression of GLUT from the class I and III was measured in the whole lung by Western blotting. Pulmonary protein content of GLUT-2, -4, -10, and -12 was significantly reduced in obese T2Dx mice (*p* = 0.032, *p* = 0.001, *p* = 0.012, and *p* = 0.034, respectively, Figure 3 and Figure 4). The pulmonary protein expression of GLUT1, one of the most broadly expressed basal transporters, and GLUT8, a novel protein isoform, was not affected by diabetes and obesity (Figure 3A and Figure 4A). Metformin treatment for 8 weeks rescued the decreased protein expression of GLUT-2, -4, and -10 in the lung of obese T2Dx mice (Figure 3B,C and Figure 4B), but not of GLUT-12 (Figure 4C). As expected, metformin treatment had no effect on pulmonary GLUT expression in healthy mice.

Immunohistochemistry experiments confirmed that GLUT4 protein was expressed in the lung and appeared to be primarily localized to the bronchial epithelium. Immunohistochemistry further indicated that inflammatory cells were only present in the untreated obese T2Dx mice, suggesting a state of inflammation in the diabetic lung (Figure S1).

### 3.4. Cell-Surface GLUT Protein Expression in the Lung of Type 2 Diabetic Mice

**Figure 5:**
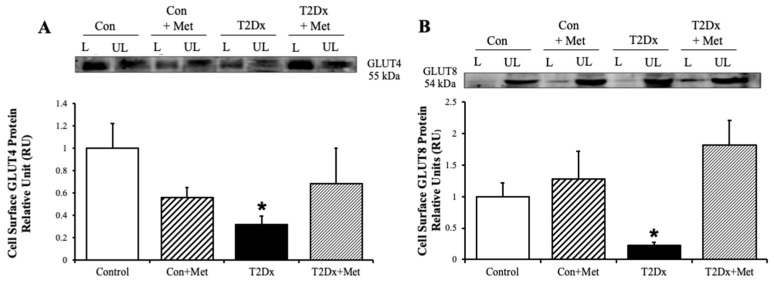
Type 2 diabetes (T2Dx) decreased pulmonary cell surface glucose transporter (GLUT) protein expression, which was rescued by in vivo metformin (Met) treatment. Cell-surface protein expression of (**A**) GLUT4 and (**B**) GLUT8 in whole lung of control (Con), untreated T2Dx, and metformin-treated T2Dx animals. Top panels: representative Western blot. Bottom panels: Mean ± SE of cell surface GLUT protein content (values normalized to respective controls, n = 4/group). L: Labeled (cell surface fraction), UL: unlabeled (intracellular fraction). * *p* < 0.05 vs. control, via one-way ANOVA. Methods: biotinylated photolabeling assay.

To determine whether obesity and diabetes alter GLUT translocation in addition to its protein expression, GLUT trafficking was evaluated in mouse whole lung via the biotinylated photolabeling technique. Cell surface protein expression of the predominant insulin-sensitive GLUT, namely GLUT4, and the novel insulin-sensitive GLUT8, were significantly decreased in the lung of obese T2Dx mice (*p* = 0.033 and *p* = 0.029 vs. control, respectively, Figure 5). Alterations in GLUT-4 and- 12 trafficking were rescued in the lung of obese T2Dx mice following long-term treatment with metformin. As expected, metformin treatment had no effect on pulmonary GLUT trafficking in healthy mice.

## 4. Discussion

Our study demonstrates that obesity and hyperglycemia during type 2 diabetes significantly altered GLUT protein expression and trafficking in the adult lung and that long-term treatment with metformin partially rescued these alterations. Consistent with epidemiologic data linking diabetes to increased respiratory infections [3,4,5,20], we found that hyperglycemia in T2Dx mice was associated with a decrease in protein levels of GLUT-2, -4, -10, and -12, as well as decreased trafficking of insulin-sensitive GLUTs in the lung. In addition, long-term treatment with metformin rescues ASL glucose levels and the expression and trafficking of several major GLUT isoforms during T2Dx, suggesting a restoration of pulmonary glucose homeostasis.

Metabolic diseases represent a critical health burden worldwide, leading to substantial morbidity, mortality, and healthcare costs. For instance, ~2.1 billion people (nearly 30 % of the global population) are overweight or obese [1,2]. In the current study, we induced T2Dx by feeding a diet with 60% kilocalories from fat for 4 months, producing very mild hyperglycemia but marked obesity and hyperinsulinemia (as determined via intraperitoneal insulin tolerance test and fasting serum insulin ELISA) [13]. We also noticed a correlation between glucose concentration in the blood and BAL fluid, suggesting that hyperglycemia leads to increased glucose concentration in ASL, which was rescued by metformin treatment. Airway glucose homeostasis is critical for immune defense and the observed elevated airway glucose levels could promote bacterial growth and lead to the formation of advanced glycation end products that have proinflammatory effects [7,9,13].

In mammals, the GLUT family comprises 14 isoforms divided into three classes based on sequence similarity [10]. Class I glucose transporters (GLUT-1, -2, -3, and -4) are the most prominent and widely expressed, while Class III transporters (GLUT-8, -10, and -12) are newer isoforms that remain less well characterized. Notably, GLUT1 is a ubiquitously expressed, membrane-bound transporter that facilitates basal glucose uptake into cells throughout the body [10,11,12,13,14,15]. GLUT2 generally facilitates bidirectional glucose transport in liver, kidney, and pancreatic beta islet cells, playing a key role in glucose sensing and homeostasis. Other GLUTs, such as GLUT4, require activation by insulin and insulin-independent (e.g., calcium) pathways to translocate from an intracellular pool to the cell surface in order to enhance glucose transport into striated muscle and adipose tissues [10,11,12,13,14,15]. The Class III GLUTs, including GLUTs -8, -10, and -12, are less well-characterized but are expressed in many tissues. For instance, GLUT8 have been reported to be expressed in the brain, testes, placenta, liver, kidney, intestine, adipose, striated muscle, and lung tissues; while GLUT12 has been reported to be expressed in striated muscle, lung, prostate, adipose, placenta and intestine tissues [9,15,16,17]. The class III GLUTs modulate glucose transport and immunometabolism and have been identified in the pathophysiology of many diseases, such as diabetes, Alzheimer’s and cardiovascular diseases [9,15,16,17].

Importantly, the P.I.’s laboratory has recently characterized the presence of several GLUT isoforms (including novel class III isoforms) in the adult lung of healthy mice, as they more likely regulate respiratory glucose homeostasis, as in other tissues [15]. In the present study, T2Dx causes significant alterations in pulmonary GLUT protein expression from the class I (GLUTs-2, -4) and class III (GLUT10) isoforms. These data suggested that T2Dx induces alteration in whole-body glucose homeostasis, including in ASL and the lung.

Glucose uptake is in part regulated by GLUT translocation from an intracellular (inactive) pool to the cell surface (active site) by insulin dependent signaling pathway. As a result, a chronic hyperinsulinemic state during T2Dx leads to alterations in the insulin signaling pathways regulating GLUT4 translocation [11,13,16]. Therefore, in the present study, we used our well-established biotinylation photolabeling assay, adapted for lung tissue, to directly and quantitatively measure active cell surface of several GLUT isoforms, providing insight into GLUT trafficking [11,12,13,14,15,16,17]. Because GLUT-1, -2, -10, and -12 predominantly reside on the cell surface and do not depend on trafficking mechanisms, we did not assess trafficking for these isoforms [10,12,17]. In contrast, GLUT4, the primary insulin-sensitive isoform, and GLUT8, a novel Class III isoform, require trafficking from intracellular pools to the cell surface upon stimulation by insulin or calcium to enhance glucose uptake into insulin-sensitive tissues [10,11,13,14,15]. Importantly, in the present study, our photolabeling data revealed a significant decrease in the cell surface expression of GLUT-4 and -8 in diabetic lungs, despite no change in total GLUT8 protein, highlighting a defect in GLUT trafficking. This mirrors observations in insulin-resistant skeletal muscle, heart, and adipose tissue, where defective GLUT4 trafficking underlies impaired glucose uptake [11,13,16]. Similarly, we previously demonstrated altered GLUT-4 and -8 trafficking to the cell surface in the cardiorespiratory system of insulin-deficient diabetic mice [11,15]. Taken together, these studies demonstrate that insulin resistance during T2Dx impaired GLUT4 trafficking to the cell surface not only in insulin-sensitive tissue but also in the lung. While the importance of GLUT8 in the lung has yet to be determined, it is worth noting that total GLUT8 protein expression was unchanged between groups despite the significant downregulation of GLUT8 at the pulmonary cell surface in diabetic subjects. These data suggest that impaired GLUT8 trafficking, rather than expression, contributes to the alteration of glucose uptake in the lung of obese diabetic mice. Thus, decreased GLUT expression and/or trafficking in the lung during diabetes likely contribute to elevated airway glucose levels and could compromise lung immunity.

Cell-specific expression of glucose transporters in the lung is not yet well understood. In the present study, immunohistochemistry was performed in the lungs of healthy, treated and untreated diabetic mice to provide some insights into GLUT4 localization in the lung. Our immunohistochemistry experiments further indicated a state of inflammation in the lung of untreated obese diabetic mice, which was not observed after long-term metformin treatment. Additional studies to further explore regional heterogenicity of the GLUTs in the lung will be required.

We also wanted to determine if common metabolic treatments already readily used in human patients, such as metformin, were effective in rescuing alterations of pulmonary glucose homeostasis in obese diabetic mice. As in humans, long treatment with metformin rescued the mild hyperglycemia of T2Dx mice. Metformin further reduced the increased glucose levels observed in the airway of obese T2Dx mice. Metformin treatment did not affect the weight of control or obese T2Dx mice. As expected, metformin treatment did not alter glucose levels in blood or BAL fluid, nor the protein expression of any GLUTs in the lung of healthy (control) mice. In contrast, metformin treatment did rescue the expression of GLUT-2, -4, and -10 in the lung of obese T2Dx mice. However, metformin did not rescue pulmonary GLUT12 expression, suggesting selective sensitivity of specific GLUT isoforms to metabolic correction in obese T2Dx mice.

Here, we also assess GLUT trafficking in the lungs of untreated and treated type 2 diabetic mice to assess by using the biotinylation photolabeling assay. Metformin treatment did not alter GLUT trafficking to the cell surface in control mice but rescued the downregulation of cell surface protein content in the lung of obese T2Dx mice, suggesting that metformin enhances pulmonary glucose transport. Metformin’s ability to rescue pulmonary GLUT-4 and -8 surface expression in T2Dx mice suggests a restoration of trafficking pathways, potentially via activation of AMPK signaling, known to promote GLUT4 translocation and thus glucose uptake in other tissues [18,19].

Metformin, an AMPK agonist, is widely used to treat T2Dx and obesity [18]. Although studies in pulmonary cells are limited, metformin has been shown to activate AMPK and reduce inflammation in lung models [21,22,23,24]. It inhibits tumor growth via an mTOR/AMPK-dependent pathway in cell cultures, mice, and humans [21,22,23]. It has also been shown to reduce lung cancer incidence [25,26]. However, its mechanisms of action, especially as they relate to noncancerous pulmonary GLUTs, are unknown. Notably, while metformin treatment did not activate AMPK in some rodent lung cancer models [22], another study reported anti-inflammatory effects during pulmonary LPS challenge through increased AMPK phosphorylation [24]. Metformin may also restore pulmonary glucose homeostasis by reducing the permeability of the tight junctions [9]. In addition, its glucose-lowering and insulin-sensitizing effects are due in part by enhancing GLUT4 translocation to the cell surface in insulin-sensitive tissues and by decreasing gluconeogenesis in the liver [27]. In the present study, metformin treatment for 8 weeks rescued pulmonary GLUT protein expression and trafficking and reduced glucose levels in the airway of obese T2Dx mice. Overall, long-term treatment with metformin not only restored whole-body but also pulmonary and airway glucose homeostasis during type 2 diabetes.

This study is robust for its use of an adult mammal model of obesity and TD2x (with a sample size sufficient to detect statistical differences for both in vivo and in vitro parameters), its use of long-term metformin treatment, and its state-of-the art photolabeled biotinylation technique to assess GLUT trafficking in the lung. However, future experiments, including methods of diabetes induction and other types of diabetes treatment would expand the conclusions drawn here. Since the majority of the population is affected by T2Dx [1,2], it is also important to understand which pathophysiological alterations are due to hyperglycemia alone (i.e., type 1 diabetes) versus those due to obesity, hyperinsulinemia and inflammation. Most importantly, while C57Bl/6 mice are an excellent and well-reported translational mammal model for human biology, including lung biology and glucose regulation [28,29], this study warrants follow-up studies using human tissues to evaluate the translational significance of the present findings. This study could also be further expanded by investigating the alterations of the downstream signaling effectors regulating glucose transport during T2Dx (e.g., insulin receptor substrate, Akt, AS160), as well as the mechanisms of action of metformin treatment in the lung [30].

## 5. Conclusions

Type 2 diabetes and obesity significantly disrupt pulmonary glucose homeostasis by decreasing expression and trafficking of glucose transporters from Class I and III, which could contribute to increase ASL glucose levels and respiratory infections. Metformin treatment rescues many of these defects, supporting its potential role not only in systemic glucose regulation but also in preserving pulmonary glucose homeostasis. Insights gained from this study could lead to the identification of metabolic therapeutic strategies, such as metformin, for obese and diabetic patients affected by concurrent respiratory infections. Further studies are warranted to elucidate the precise mechanisms by which type 2 diabetes and metformin modulate lung glucose transport and to explore their impact on susceptibility to respiratory infections.

## Data Availability

The original contributions presented in this study are included in the article.

## Supplementary Materials

The following supporting information can be downloaded at: https://www.mdpi.com/article/10.3390/metabo15110717/s1, Figure S1: Immunohistochemical GLUT-4 staining in the lung. Representative images (n = 3/group) from healthy (A), untreated T2Dx (B), and T2Dx mice treated with metformin (C). Brown color indicates positive result, purple color indicates negative result (negative control, D). Structures are labeled as: black star: bronchiole, white star: alveoli, white arrow: blood cells in pulmonary vessel, black arrow: inflammatory cells. Scale bar = 50 µm.
