# Supplementary figures and images for: Altered Pulmonary Glucose Transport Is Restored by Metformin Treatment in an Obese Type 2 Diabetic Animal Model
Source: Metabolites. 2025 Nov 2;15(11):717. doi: 10.3390/metabo15110717 (PMC12654456; doi:10.3390/metabo15110717)

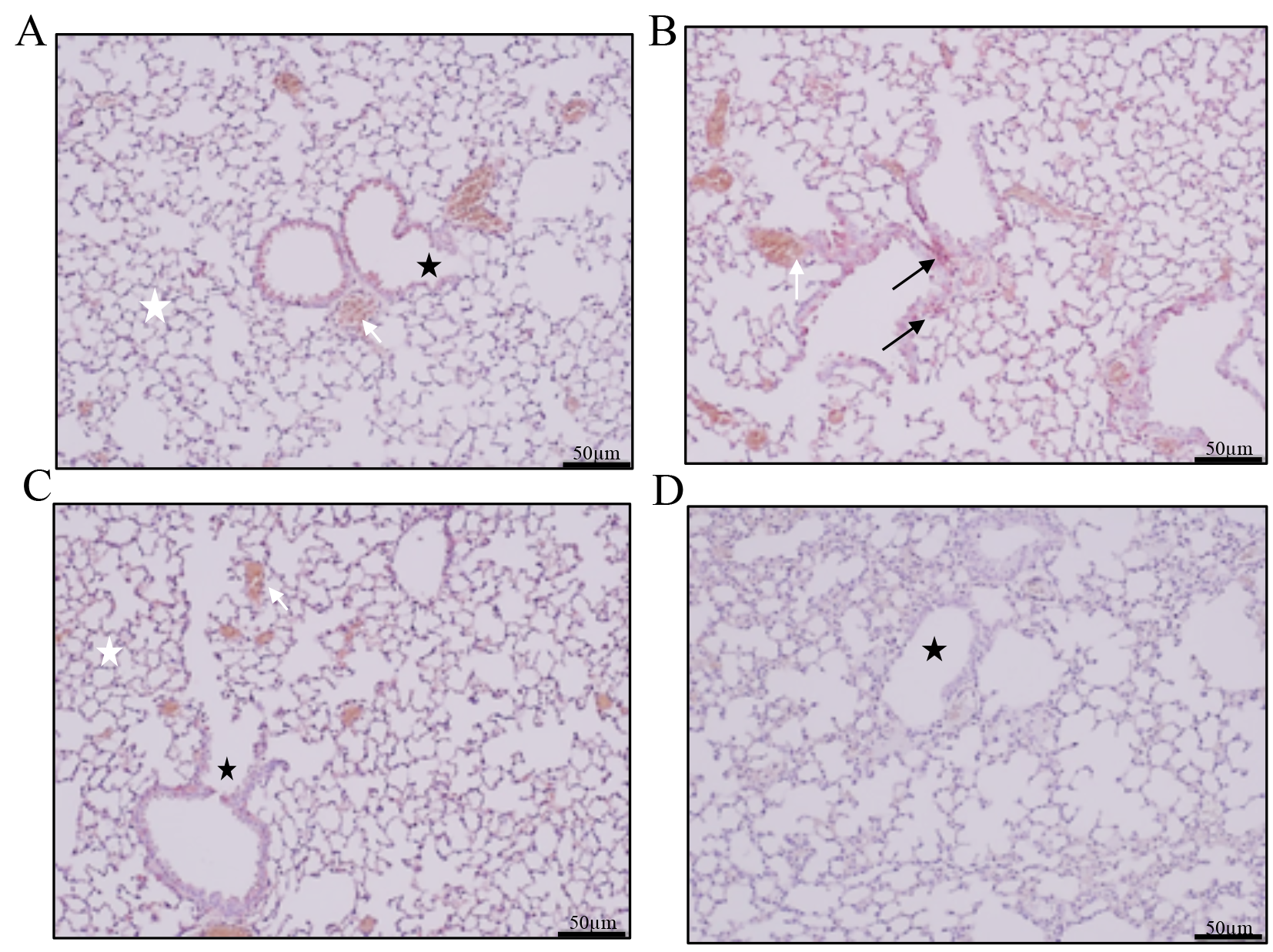

Supplement: Supplementary file 1 [file metabolites-15-00717-s001.zip › metabolites-3760678-supplementary.tiff]
